#  The role of ncRNA regulatory mechanisms in diseases—case on gestational diabetes

**DOI:** 10.1093/bib/bbad489

**Published:** 2024-01-06

**Authors:** Dong Gao, Liping Ren, Yu-Duo Hao, Nalini Schaduangrat, Xiao-Wei Liu, Shi-Shi Yuan, Yu-He Yang, Yan Wang, Watshara Shoombuatong, Hui Ding

**Affiliations:** School of Life Science and Technology, Center for Informational Biology, University of Electronic Science and Technology of China, Chengdu 610054, China; School of Healthcare Technology, Chengdu Neusoft University, Chengdu 611844, China; School of Life Science and Technology, Center for Informational Biology, University of Electronic Science and Technology of China, Chengdu 610054, China; Center for Research Innovation and Biomedical Informatics, Faculty of Medical Technology, Mahidol University, Bangkok 10700, Thailand; School of Life Science and Technology, Center for Informational Biology, University of Electronic Science and Technology of China, Chengdu 610054, China; School of Life Science and Technology, Center for Informational Biology, University of Electronic Science and Technology of China, Chengdu 610054, China; School of Life Science and Technology, Center for Informational Biology, University of Electronic Science and Technology of China, Chengdu 610054, China; Department of Cardiovascular Medicine, Beijing Hospital, National Center of Gerontology, Institute of Geriatric Medicine, Chinese Academy of Medical Sciences, Beijing 100730, China; Center for Research Innovation and Biomedical Informatics, Faculty of Medical Technology, Mahidol University, Bangkok 10700, Thailand; School of Life Science and Technology, Center for Informational Biology, University of Electronic Science and Technology of China, Chengdu 610054, China

**Keywords:** non-coding RNA, gestational diabetes, regulatory mechanism, biomarkers, attention mechanism

## Abstract

Non-coding RNAs (ncRNAs) are a class of RNA molecules that do not have the potential to encode proteins. Meanwhile, they can occupy a significant portion of the human genome and participate in gene expression regulation through various mechanisms. Gestational diabetes mellitus (GDM) is a pathologic condition of carbohydrate intolerance that begins or is first detected during pregnancy, making it one of the most common pregnancy complications. Although the exact pathogenesis of GDM remains unclear, several recent studies have shown that ncRNAs play a crucial regulatory role in GDM. Herein, we present a comprehensive review on the multiple mechanisms of ncRNAs in GDM along with their potential role as biomarkers. In addition, we investigate the contribution of deep learning-based models in discovering disease-specific ncRNA biomarkers and elucidate the underlying mechanisms of ncRNA. This might assist community-wide efforts to obtain insights into the regulatory mechanisms of ncRNAs in disease and guide a novel approach for early diagnosis and treatment of disease.

## INTRODUCTION

Approximately 75% of human genes are transcribed into RNA, but only 3% of these transcripts are translated into protein-coding mRNAs [1]. The remaining non-coding RNAs (ncRNAs) are a class of RNA molecules that do not have the potential to code for proteins; instead, they play crucial roles in various biological processes directly through their transcripts [2]. Common types of ncRNAs include microRNAs (miRNAs), long non-coding RNAs (lncRNAs) and circular RNAs (circRNAs). With the development of high-throughput sequencing technology and bioinformatics, numerous studies have shown that ncRNAs play critical roles in the occurrence and development of various types of diseases, such as cardiovascular disease, cancers and diabetes. This has led to the significant potential of ncRNAs as diagnostic biomarkers and therapeutic targets, particularly miRNAs, which have been demonstrated to be critical regulatory factors in cardiovascular risk and cellular responses. For instance, in heart failure, miRNAs may regulate pathways such as cardiac hypertrophy, inflammatory response, regeneration and angiogenesis through changes in their own expression or by binding to target mRNAs [3]. Recent studies have also indicated promising prospects for small, non-coding nucleic acid therapeutics in the clinical treatment of cardiovascular diseases [4]. These findings suggest that ncRNAs have great potential in diagnosing and treating diseases. Researching their expression and molecular mechanisms is beneficial for deepening our understanding of disease occurrence and development.

Gestational diabetes mellitus (GDM) is currently one of the most common complications during pregnancy, characterized by the development of chronic insulin resistance and high blood glucose levels occurring in the mid to late second trimester (weeks 13–26) or early third trimester (weeks 27–40) of pregnancy [5, 6]. Although hyperglycemia subsides after delivery, GDM may cause various complications during pregnancy and delivery. These symptoms can have short-term and even long-term adverse effects on the mother, fetus and offspring. Short term, women who develop GDM have a higher risk of adverse pregnancy outcomes including gestational hypertension, preeclampsia and premature delivery [7, 8]. Long term, women with a history of GDM and their offspring are prone to metabolic disorders and have an increased risk of developing conditions such as type 2 diabetes mellitus and cardiovascular disease [9–11]. The prevalence of GDM is on the rise worldwide [12]. However, due to variations in GDM screening and diagnostic criteria among countries, the recorded prevalence of GDM varies significantly, ranging from 1% to >30% [5]. Using the International Association of the Diabetes and Pregnancy Study Groups (IADPSG) criteria, the prevalence of GDM was 17.8% (range 9–26%) in a multinational cohort of women across 15 centers in the Hyperglycemia and Adverse Pregnancy Outcome (HAPO) study [13]. Similarly, when applying the IADPSG diagnostic criteria, a systematic review and meta-analysis that included 25 cross-sectional or retrospective studies conducted in mainland China, involving 79 064 Chinese participants, revealed a high overall incidence rate of GDM among Chinese women, reaching 14.8% [14]. Given the various adverse effects of GDM on the health outcomes of both maternal and offspring, as well as the increasing incidence rates, it is crucial to elucidate the pathogenesis of GDM and identify effective biomarkers for early prevention, risk assessment, disease diagnosis and targeted therapies.

Currently, treatment strategies for GDM mainly include insulin therapy, metformin therapy, probiotic supplementation and vitamin D supplementation [15]. It has been shown that metformin treatment alters the expression levels of ncRNAs in diabetes mellitus [16]. As important participants in metabolic processes, there is a significant association between altered expression of ncRNAs and GDM. Du et al. reviewed the role of aberrantly expressed ncRNAs in the placenta in GDM and pregnancy-related complications. They found that these ncRNAs are associated with abnormal placental structures, metabolic disturbances and pathological features of GDM. In addition, placenta-specific ncRNAs may serve as potential diagnostic biomarkers and therapeutic targets for GDM [17]. However, the exact mechanisms of ncRNAs in the development of GDM and their potential as therapeutic targets are not entirely understood. Thus, identifying ncRNA biomarkers and understanding their functions might contribute to elucidating the complex pathophysiological mechanisms in GDM. This, in turn, could help improve early prevention and diagnosis of GDM, enabling early intervention and personalized medicine for GDM patients. Such advancements are of significant importance in enhancing the health status of both pregnant women and their offspring.

In this review, we summarize recent research on the dysregulated regulatory mechanisms of ncRNAs in GDM and offer crucial suggestions for utilizing deep learning models to discover new biomarkers and predict potential mechanisms in the future.

## BIOGENESIS OF NCRNAS

### Long non-coding RNAs

**Figure 1:**
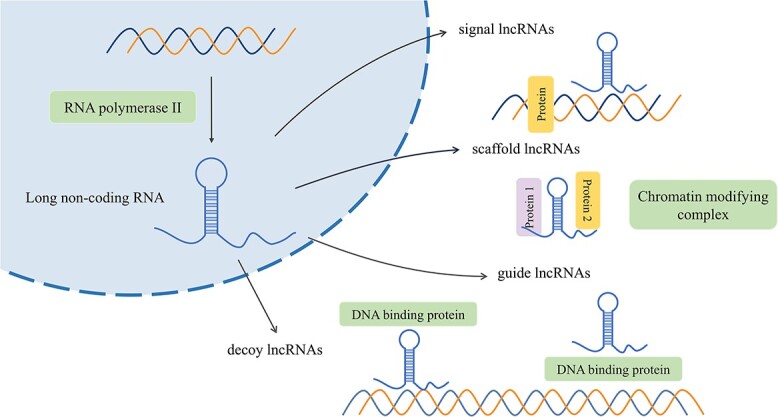
The biogenesis of lncRNAs.

Long non-coding RNAs are transcripts longer than 200 nucleotides (nt) that do not encode proteins. They are mainly located in less conserved regions of the genome [18]. Most lncRNAs are transcribed by RNA Polymerase II (RNAP2) in the nucleus from intergenic regions, within introns of protein-coding genes, or the antisense strand of genes. To enhance their stability, primary transcripts are generally 3′-polyadenylated, 5′-capped and alternatively spliced [19, 20], resulting in mRNA-like characteristics. In addition, they can also be transcribed from conserved genomic regions and reverse splicing of exons [21]. The final step in lncRNA biogenesis is the formation of thermodynamically stable structures that allow them to interact with DNA, RNA and proteins to exert their functions [22]. Based on these interactions, lncRNAs can be classified into four categories: signal lncRNAs, decoy lncRNAs, guide lncRNAs and scaffold lncRNAs [23] (Figure 1). They can mediate various biological processes through a variety of mechanisms, i.e. transcriptional interference, induction of chromatin remodeling, regulation of alternative splicing patterns, modulation of protein activity and alteration of protein localization within the cell. For example, lncRNAs can act as *cis*-acting elements (*cis*) to regulate the expression of protein-coding genes in the proximity of their own expression sites [24]. Some lncRNAs can also function as miRNA sponges, known as competitive endogenous RNAs (ceRNAs). By binding to one or multiple miRNAs, they titrate miRNAs away from their target genes, thereby regulating miRNA-mediated post-transcriptional silencing [25]. These mechanisms highlight the vital role of lncRNAs in various biological processes, and dysregulation of lncRNA expression may result in the progression of various diseases, including GDM.

### MicroRNAs

**Figure 2:**
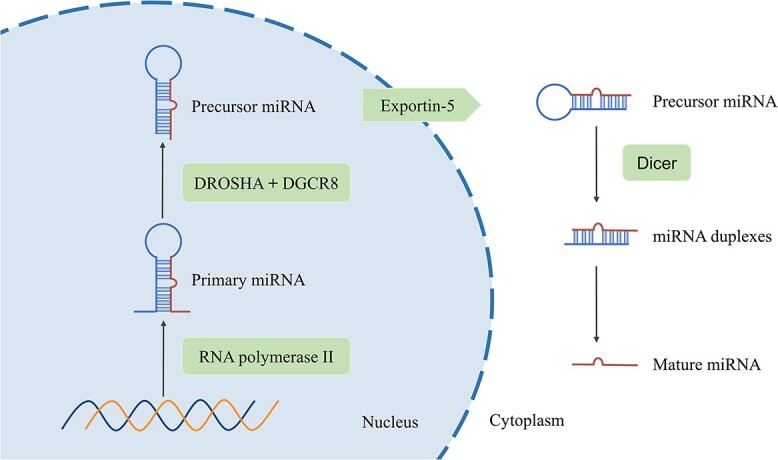
The biogenesis of miRNAs.

miRNAs are highly conserved non-coding RNA sequences consisting of ~22 nt. In the nucleus, miRNAs are primarily transcribed by RNA polymerase II, producing a primary miRNA (pri-miRNA) of ~500–3000 nt in length [26]. Then, the pri-miRNA is processed by the microprocessor complex, which contains the endoribonuclease DROSHA and its RNA-binding partner DGCR8, to generate an ~70 nt precursor miRNA (pre-miRNA) [27]. The pre-miRNA is then exported to the cytoplasm via the nuclear export protein Exportin-5. In the cytoplasm, the pre-miRNA is further sheared into a 22 nt double-stranded miRNA duplexes by Dicer. In the double-stranded structure, the guide RNA strand becomes the mature miRNA, while the passenger strand is degraded [28] (Figure 2). miRNAs typically regulate gene expression post-transcriptionally by directly interacting with partially or fully complementary target sites in mRNA 3′ untranslated region (3′-UTR), 5′ untranslated region (5′-UTR) or open reading frames, inhibiting mRNA translation or causing its degradation [29, 30]. It is worth noting that over 60% of human mRNAs contain target sites for miRNAs, and a single miRNA can regulate the expression of multiple target genes, while one gene can also be regulated by multiple miRNAs [31]. Substantial research has shown that miRNAs play a regulatory role in various biological processes, such as cell proliferation, differentiation and development [32]. Moreover, dysregulation of their expression is closely associated with diseases like cancer, cardiovascular disorders and GDM. This underscores the great potential of miRNAs as disease biomarkers and therapeutic targets.

### Circular RNAs

**Figure 3:**
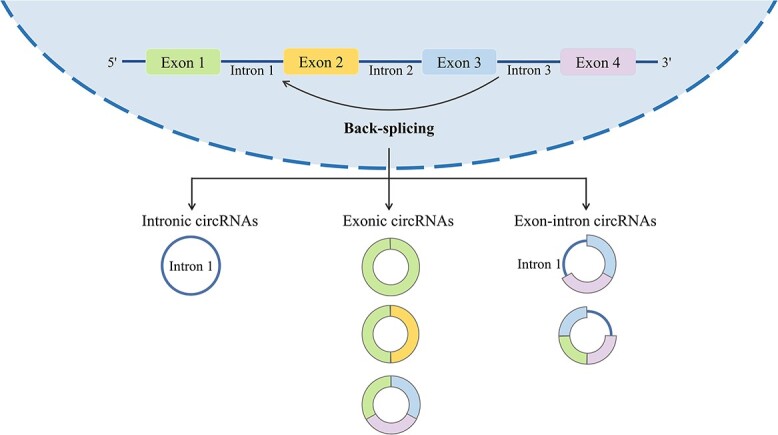
The biogenesis of circRNAs.

Unlike linear RNAs such as lncRNAs and miRNAs, circRNA is a covalently closed, circular single-stranded RNA that typically lacks the ability to code for proteins. Most splice sites downstream are connected to 3′ splice site upstream. This process leads to the formation of a circular RNA molecule with a 3′–5′ phosphodiester bond at the back-splicing junction site [33]. Owing to the absence of 5′ caps and 3′ poly-A tails, circRNAs are immune to RNA nucleic acid exonucleases, making their expression more stable and less susceptible to degradation [34]. However, since the efficiency of reverse splicing is much lower than that of conventional splicing, the abundance of circRNAs is generally lower in cells and tissues. Based on their composition, circRNAs can be categorized into several types: exonic circRNAs (ecircRNAs)—formed by exons; intronic circRNAs (ciRNAs)—formed by introns; exon–intron circRNAs (EIciRNAs)—formed by both exons and introns [35] (Figure 3). They typically function at the transcriptional or post-transcriptional level. For example, circRNAs can act as miRNA sponges, inhibiting miRNA binding to the non-coding region of target genes to regulate gene expression. They also interact with transcription factors to influence gene transcription. In addition, circRNAs interfere with the normal splicing of mRNA precursors, regulate mRNA translation, form circular RNA–protein complexes and compete with mRNA-binding proteins, participating in biological processes such as immunity, metabolism and neural system development [33]. Some studies have shown that circRNAs play crucial roles in the initiation and development of diseases, especially in conditions like GDM, where they may affect maternal blood glucose levels by regulating beta-cell proliferation and glucose metabolism. Modulating circRNA expression levels to maintain normal cellular function could potentially offer a novel approach to treating GDM.

## ROLE OF NCRNAS IN GDM

### Long non-coding RNAs

The abnormal expression of lncRNAs in the plasma, serum and placental tissues of GDM patients is closely associated with the occurrence of GDM and its complications. Therefore, studying the expression profiles of lncRNAs in different tissues of GDM patients is of crucial significance (Table 1). For instance, in the plasma of GDM women during the first and second trimesters, the expression levels of NONHSAT054669.2 and ENST00000525337 were significantly higher than pregnant women with normal glucose tolerance (NGT). In addition, the expression level of NONHSAT054669.2 was positively correlated with the oral glucose tolerance test levels [36]. This suggests that these lncRNAs have a higher diagnostic value for GDM in the first and second trimesters. In serum, the Pearson correlation coefficient showed that a high lncRNA HOTAIR expression level positively correlated with body mass index, fasting plasma glucose, 1 h plasma glucose and 2 h plasma glucose in GDM patients. Therefore, it can be used as a diagnostic marker for GDM [37]. lncRNA SOX2OT is also highly expressed in GDM and strongly associated with multiple adverse events [38]. Furthermore, expression levels of lncRNAs can predict the occurrence of GDM-related adverse effects, such as kidney injury, preterm labor and macrosomia. In a 6 year follow-up study of 400 women with planned pregnancies, it was found that patients with higher plasma levels of lncRNA MEG8 prior to pregnancy had a higher incidence of GDM during pregnancy. Furthermore, GDM patients with higher MEG8 levels at discharge also had a significantly increased risk of kidney injury [39]. The lncRNA SNHG17 is expressed at lower levels in the plasma of pregnant women with GDM. This lower expression may contribute to the occurrence of GDM by inhibiting the growth of INS-1 cells and reducing insulin secretion by these cells. Furthermore, these levels are highly correlated with adverse perinatal outcomes, such as preterm labor [40].

**Table 1:** Summary and functions of aberrant lncRNAs in GDM

lncRNAs	Expression in GDM	Target	Function	Source	Groups		Species	Ref.
lncRNA HCG27	Down	miR-378a-3p	HCG27 enhances glucose uptake in HUVECs through the miR-378a-3p/MAPK1 signaling pathway	Placenta HUVECs	25 GDM	25 NGT	*Homo sapiens*	[45]
lncRNA TUG1	Down	miR-328-3p	TUG1 prevents IR through competitively binding to miR-328-3p and promoting the SREBP-2-mediated ERK signaling pathway inactivation	Islet tissue Blood from eyeballs	100 GDM	20 NGT	Mouse	[44]
ENONHSAT054669.2 ENST00000525337	**First trimester** Up	–	Novel diagnostic biomarkers for early diagnosis of GDM	Plasma	**First trimester**		*Homo sapiens*	[36]
					27 GDM	45 NGT		
					**Second trimeste** **r**			
					56 GDM	58 NGT		
					**Third trimester**			
					39 GDM	37 NGT		
CCDC144NL-AS1	Up	miR-143-3p	CCDC144NL-AS1 controls trophoblast cell development by negatively regulating miR-143-3p	Placenta Serum	138 GDM	102 NGT	*Homo sapiens*	[46]
lncRNA SNX17	Up	miR-517a	lncRNA SNX17 promotes the trophoblast proliferation through miR-517a/IGF-1 pathway	Placenta	–		*Homo sapiens*	[48]
LINC00667 LINC01087 AP000350.6 CARMN	–	–	A m6A-related subnetwork is constructed using these four lncRNAs, their ceRNAs, and associated m6A regulators	Placenta	**Discovery**		*Homo sapiens*	[53]
					32 GDM	32 NGT		
					**Validation**			
					GSE2956			
					3 GDM	3 NGT		
					GSE19649			
					1 GDM	1 NGT		
					GSE70493			
					3 GDM	3 NGT		
lncRNA HOXA	Down	miR-423-5p	HOTTIP ameliorates IR and hepatic gluconeogenesis via the modulation of the miR-423-5p/WNT7A axis	Pancreatic cells	10 Groups 6 mice/group		Mouse	[43]
lncRNA MEG8	Up	miR-296-3p	MEG8-siRNA promotes pancreatic β-cell functions by targeting miR-296-3p	Peripheral blood	30 GDM	30 NGT	*Homo sapiens*	[42]
lncRNA MEG8	Up	–	Plasma levels of MEG8 can predict GDM among pregnant females	Plasma	78 GDM	322 NGT	*Homo sapiens*	[39]
lncRNA HOTAIR	Up	–	The abnormal expression of HOTAIR may play a role in GDM occurrence	Serum	99 GDM	98 NGT	*Homo sapiens*	[37]
lncRNA SOX2OT	Up	–	SOX2OT is highly expressed in GDM and closely correlated with multiple adverse events	Plasma	108 High SOX2OT 108 Low SOX2OT		*Homo sapiens*	[38]
lncRNA XIST	Up	miR-497-5p	Inhibition of XIST might alleviate the adverse function of HG on cell viability via sponging miR-497-5p	Serum HTR-8/SVneo cells	93 GDM	93 NGT	*Homo sapiens*	[47]
lncRNA OIP5-AS1	Down	miR-137-3p	Upregulation of OIP5-AS1 ameliorates cell injury partially by sponging miR-137-3p	Serum HTR-8/SVneo cells	75 GDM	75 NGT	*Homo sapiens*	[50]
lncRNA RPL13P5 lncRNA ERMP1 lncRNA TSPAN32 lncRNA MRPL38	Up Up Up Up	TSC2 TPH1	lncRNAs RPL13P5, ERMP1, TSPAN32 and MRPL38 form a co-expression network with target genes	Plasma	25 GDM	19 NGT	*Homo sapiens*	[52]
lncRNA RPL13p5	Up	TSC2	lncRNA RPL13p5 forms a co-expression strand with the TSC2 gene via the PI3K-AKT signaling pathway	Peripheral blood	25 GDM	19 NGT	*Homo sapiens*	[51]
lncRNA SNHG17	Down	–	Low plasma expression levels of SNHG17 is correlated with the high incidence rate of GDM	**Discovery** Plasma **Validation** Plasma	**Discovery**		*Homo sapiens*	[40]
					60 GDM	60 NGT		
					**Validation**			
					240 Pregnant females			
lncRNA MALAT1	Up	TGF-β/NF-κB pathway	Downregulation of lncRNA MALAT1 inhibits inflammation and suppresses the proliferation possibly by modulating the TGF-β/NF-κB signaling pathway	Placental HTR8 cells	78 GDM	38 NGT	*Homo sapiens*	[49]

lncRNAs, long non-coding RNAs; GDM, gestational diabetes mellitus; NGT, normal glucose tolerance; HUVECs, human umbilical vein endothelial cells; IR, insulin resistance; ceRNAs, competing endogenous RNAs; High SOX2OT, high expression level of SOX2OT; Low SOX2OT, low expression level of SOX2OT; HG, high glucose.

The alteration in the expression levels of lncRNAs has shown great potential in the discovery of early diagnostic and therapeutic biomarkers for GDM, providing new insights into the prevention and mitigation of adverse reactions associated with GDM. Therefore, exploring their regulatory mechanisms in the development of GDM is of great significance in guiding the early prevention, diagnosis and treatment of GDM (Table 1). The development of GDM involves various mechanisms, including β-cell dysfunction, insulin resistance (IR), adipose tissue dysfunction, gluconeogenesis and oxidative stress [41]. Several studies have shown that lncRNAs can be involved in the development of GDM by targeting genes, pathways or by acting as ceRNAs. β-Cell dysfunction is a major pathophysiological feature of GDM. Similar to the findings of a previous study [39], lncRNA MEG8 is highly expressed in patients with GDM. This high expression may lead to β-cell dysfunction through negative regulation of miR-296-3p, ultimately suppressing insulin secretion [42]. In a study on GDM mice, researchers found decreased expression levels of lncRNA HOXA transcript at the distal tip (HOTTIP) and WNT7A, while the level of miR-423-5p was increased. The overexpression of HOTTIP could alleviate insulin resistance and hepatic gluconeogenesis in GDM mice by regulating the miR-423-5p/WNT7A axis [43]. Another study of insulin resistance in GDM mice has shown that lncRNA TUG1 could prevent IR after GDM by competitively binding to miR-328-3p and promoting SREBP-2-mediated inactivation of the ERK signaling pathway [44]. In umbilical vein endothelial cells (HUVECs) of GDM patients, the expression of lncRNA HCG27 is significantly decreased, while miR-378a-3p is significantly increased, and MAPK1 expression is reduced. LncRNA HCG27 might promote glucose uptake of HUVECs by miR-378a-3p/MAPK1 pathway [45]. Trophoblast cells play a crucial role in maintaining pregnancy, supporting fetal growth and development, and modulating the immune response during pregnancy. Dysfunction of its cellular functions may lead to GDM. Compared to healthy pregnant women, the expression of CCDC144NL-AS1 was significantly upregulated in serum and placental tissues of patients with GDM. Also, the placenta expression level was positively correlated with the index of insulin resistance, which is a major risk factor for the onset and progression of GDM. Furthermore, CCDC144NL­AS1 may regulates the trophoblast cell proliferation, migration and invasion via miR­143-3p [46]. The expression of lncRNA XIST was increased in GDM patients and high glucose (HG) HTR-8/SVneo cell models. Inhibition of XIST might alleviate the adverse function of HG on cell viability via sponging miR-497-5p, which may target FOXO1 to mediate the occurrence of GDM [47]. In contrast to the findings of Li et al., lncRNA SNX17 was dramatically upregulated in placental tissues of patients with macrosomia and may promote trophoblast proliferation through the miR-517a/IGF-1 pathway [48]. Similarly, the expression of lncRNA MALAT1 was higher in the placental tissues of the GDM patient group. At the molecular level, downregulation of lncRNA MALAT1 may inhibit the secretion of inflammatory factors and inhibit the proliferation, invasion and migration of GDM placental trophoblasts. This might be mediated through TGF-β and NF-κB signaling pathways [49]. In addition, decreased expression of OIP5-AS1 was demonstrated in women with GDM. Overexpression of OIP5-AS1 can ameliorate HG-induced HTR-8/SVneo cell injury in part by sponging miR-137-3p [50]. In addition to the above mechanisms, lncRNAs can interact with other RNAs to form co-expression networks and ceRNAs networks involved in GDM pathogenesis. In the peripheral blood of GDM patients, lncRNA RPL13P5 forms a co-expression chain with the TSC2 gene through the PI3K-AKT signaling pathway as part of the insulin resistance process in GDM. Similarly, the lncRNA RPL13P5 forms a co-expression network with TSC2 genes through PI3K-AKT and insulin signaling pathways, both of which are involved in insulin resistance in GDM [51]. In another study, researchers found that lncRNA ERMP1, TSPAN32 and MRPL38 form a co-expression network with TPH1 in peripheral blood samples from pregnant women with the disease. TPH1 is primarily involved in the tryptophan metabolism pathway and the development of GDM [52]. LncRNAs can interact with m6A through the ceRNAs network to mediate the onset of GDM. Du et al. constructed a ceRNAs network consisting of 16 lncRNAs, 17 miRNAs, 184 mRNAs and 338 edges to investigate this interaction. They identified four m6A-associated lncRNAs in this network. Furthermore, the genes in the m6A-related subnetwork, based on these four lncRNAs, were enriched in GDM-related hormone signaling pathways [53].

### MicroRNAs

Dysregulated miRNA expression can be used as a marker for early diagnosis of GDM (Table 2). In a study using next-generation sequencing to identify plasma miRNA markers in patients with GDM in early pregnancy, 17 miRNAs associated with GDM development were identified, which may be involved in the regulation of lipid metabolism and insulin sensitivity (IS). Among them, three miRNAs, hsa-miR-517a-3p|hsa-miR-517b-3p, hsa-miR-218-5p and hsa-let7a-3p, have comparable predictive ability to traditional GDM risk factors [54]. Another study observed that plasma miRNAs from 18 patients with early pregnancy GDM predicted IS in the late second trimester of pregnancy [55]. Similarly, the dysregulation of miRNA expression in serum samples may serve as a diagnostic marker for GDM. miR-16-5p, miR-142-3p and miR-144-3p were significantly upregulated, with a positive correlation between miR-142-3p and plasma glucose post-loading in GDM patients [56]. In a study of microRNAs in early pregnancy serum from a Malay population, researchers found significantly elevated expression levels of hsa-miR-193a, hsa-miR-21, hsa-miR-23a and hsa-miR-361, but miR-130a was significantly downregulated. These miRNAs could potentially serve as GDM biomarkers and may be involved in the pathologic process of GDM by regulating common target genes [57]. A cross-sectional study identified a total of 157 dysregulated miRNAs in placental tissues of women with GDM. miRNA-125b and miRNA-144 are consistently dysregulated and have good diagnostic value for GDM. The results of functional enrichment analysis of target genes suggest that these two miRNAs may be involved in energy metabolism and, thus, influence glucose metabolism [58]. Furthermore, in a nested case–control study of participants from the European multicenter ‘Vitamin D and lifestyle intervention for GDM prevention (DALI)’ trial, elevated expression levels of miR-16-5p, miR-29a-3p and miR-134-5p were found, and combining them into a 3-miRNA signature could effectively predict GDM [59]. Two circulating miRNA biomarkers, miR-222-3p and miR-409-3p, have also been discovered in exosomes, which improve GDM classification and may contribute to GDM through metabolic alterations [60]. In addition, miR-27a-3p expression levels in peripheral blood mononuclear cells (PBMCs) were significantly higher in GDM patients than in controls, which correlated with lipid metabolism parameters and could be used as a diagnostic marker for GDM and as a marker for assessing pregnancy-associated metabolic status [61].

**Table 2:** Summary and functions of aberrant miRNAs in GDM

miRNAs	Expression in GDM	Target	Function	Source	Groups		Species	Ref.
miR-135a-5p	Up	SIRT1 PI3K/AKT pathway	miR-135a-5p increases the proliferation, invasion and migration of placental trophoblast cells by targeting SIRT1	Placenta Serum Plasma	30 GDM	30 NGT	*Homo sapiens*	[64]
miR-196a2 miR-27a	–	–	miR-27a rs895819 is associated with increased GDM susceptibility and higher blood glucose levels	–	500 GDM	502 NGT	*Homo sapiens*	[79]
miR-195-5p	Up	VEGFA	miR-195-5p downregulation alleviated hPMECs dysfunction by upregulating VEGFA expression	hPMECs Placenta	–		*Homo sapiens* Mouse	[65]
miR-140-3p miR-574-3p	Down Down	VEGF	Exosomal miR-140-3p and miR-574-3p downregulate VEGF expression in endothelial cells, thereby inhibiting the functional capacity of umbilical vein endothelial cells	Placenta HUVECs MUVECs	–		*Homo sapiens* Mouse	[104]
miR-423-5p miR-122-5p miR-148a-3p miR-192-5p miR-99a-5p	Up Down Down Down Down	IGF1R GYS1 G6PC3 FDFT1	Dysregulated exosomal miRNAs in plasma from pregnant women with GDM might influence the insulin and AMPK signaling pathways and could contribute to the early prediction of GDM	Plasma	**Discovery** 12 GDM 12 NGT **Validation**102 GDM 102 NGT		*Homo sapiens*	[77]
								
							
								
miR-27a-3p	Up	–	miR-27a-3p serves as a diagnostic biomarker of GDM	PBMCs	42 GDM	34 NGT	*Homo sapiens*	[61]
miR-143-3p	Down	TAK1/NF-κB pathway	miR-143-3p overexpression prevents pancreatic β-cell dysfunction by inhibiting the TAK1/NF-κB pathway	Whole blood MIN6 cells	30 GDM	30 NGT	*Homo sapiens*	[70]
miR-518	Up	PPARα	miR-518 regulates the serum inflammatory factors by inhibiting PPARα	Serum	118 GDM 57 GDM and HDCP 65 NGT		*Homo sapiens*	[63]
miR-22 miR-372	Down Down	SLC2A4 PI3K/AKT/GLUT4 pathway	The downregulations of miR-22 and miR-372 contributes to GDM through regulating the PI3K/GLUT4 pathway	Placental HRT8/SVneo cells	75 GDM	75 NGT	*Homo sapiens*	[69]
17 miRNAs	–	–	hsa-miR-517a-3p|hsa-miR-517b-3p, hsa-miR-218-5p and hsa-let7a-3p combines with classic GDM risk factors provide excellent prediction values	Plasma	**Discovery** 56 GDM 387 NGT **Validation** 76 GDM 63 NGT		*Homo sapiens*	[54]
18 miRNAs	–	–	Plasma miRNAs detected between the 4th and 16th week of pregnancy can predict IS levels assessed pregnancy	Plasma	105 GDM		*Homo sapiens*	[55]
miR-16-5p miR-142-3p miR-144-3p	Up Up Up	–	miRNAs as a predictive factor could be useful in early diagnosis	Serum	24 GDM	24 NGT	*Homo sapiens*	[56]
miR-17-5p	Down	TXNIP/NLRP3 inflammasome pathway	miR-17-5p ameliorates the glucose uptake of HTR8/SVneo cells by TXNIP/NLRP3 axis	Placental HTR8/SVneo cells	16 GDM	16 NGT	*Homo sapiens*	[68]
hsa-miR-193a hsa-miR-21 hsa-miR-23a hsa-miR-361 miR-130a	Up Up Up Up Down	KLF, ZNF25, AFF4, C1orf143, SRSF2, ZNF655	hsa-miR-193a, hsa-miR-21 and miR-130a have potential biomarker features in GDM	Serum	24 GDM	24 NGT	*Homo sapiens*	[57]
miR-199a	Up	MeCP2-TRPC3 pathway	miR-199a-5p regulates the glucose pathway by inhibiting the expression of MeCP2 and downregulating canonical Trpc3	Placental	137 GDM	158 NGT	*Homo sapiens*	[78]
miR-130b-3p	Up	ICAM-1	miR-130b-3p repressed proliferation, migration and angiogenesis of HUVECs by regulating ICAM-1 expression	Placental HUVECs	20 GDM	20 NGT	*Homo sapiens* Mouse	[75]
miR-222-3p miR-409-3p	Up Up	–	miR-222-3p and miR-409-3p serves as plasma biomarkers	Plasma	**Discovery**		*Homo sapiens*	[60]
					3 GDM	3 NGT		
					**Validation**			
					12 GDM	12 NGT		
miR-210-3p	Up	Dtx1	miR-210-3p impairs pancreatic β-cell function and cell viability by inhibiting the expression of Dtx1, promoting the progression of GDM	Serum exosomes	80 GDM	10 NGT	Mouse	[82]
miR-125b miR-144	Down Up	Metabolism-related pathways Insulin signaling pathway	miR-125b affects blood glucose metabolism through energy metabolism-related pathways miR-144 participates in energy metabolism by the development of type β pancreatic cells and energy homeostasis	Placental Serum exosomes	**Discovery** 3 GDM 3 NGT **Validation**36 GDM 37 NGT57 GDM 61 NGT		*Homo sapiens*	[58]
								
							
								
								
miR-30d-5p	Down	RAB8A	miR-30d-5p affects trophoblast cell function through negative regulation of RAB8A	Placenta	166 GDM	196 NGT	*Homo sapiens*	[67]
miR-362-5p	Down	GSR PI3K/AKT pathway	miR-362-5p promotes cell proliferation and inhibits apoptosis via targeting GSR and activating PI3K/AKT pathway	Placenta HTR-8/SVneo cells HEK293T cells	40 GDM	40 NGT	*Homo sapiens*	[66]
miR-6869-5p	Down	PTPRO	miR-6869-5p is involved in maintaining placental microenvironment balance by preventing from inflammation and inducing M2 macrophages	Placenta	26 GDM	23 NGT	*Homo sapiens*	[76]
miR-574-5p miR-3135b	Down Down	–	The common target genes of miR-574-5p and miR-3135b are associated with glucose and/or lipid metabolism, and insulin secretion and signaling pathways	Plasma	53 GDM	46 GDM	*Homo sapiens*	[62]
miR-16-5p miR-29a-3p miR-134-5p	Up Up Up	–	3-miRNA signature serves as valuable predictive biomarkers of GDM	Serum	82 GDM	41 NGT	*Homo sapiens*	[59]
miR-34b-3p	Up	PDK1	miR-34b-3p impairs HUVECs viability and migration in GDM by targeting PDK1	HUVECs	20 GDM	27 NGT	*Homo sapiens*	[74]
miR-182-3p	Up	INSR1	miR-182-3p inhibitor promotes GLUT4 translocation and glucose uptake and consumption by increasing the expression of INSR1 and its downstream signaling pathways in skeletal muscle	Skeletal muscle C2C12 muscle cells	–		Mouse	[80]
miR-92a-3p	Up	JAK/STAT signaling	EV-associated miRNAs regulate protein expression in skeletal muscles, potentially influencing insulin resistance and maternal metabolism associated with GDM	Plasma EVs	**Discovery**		*Homo sapiens*	[105]
					**First trimester**			
					2 GDM	4 NGT		
					**Second trimeste** **r**			
					9 GDM	8 NGT		
					**Third trimester**			
					4 GDM	4 NGT		
					**Validation**			
					8 GDM	14 NGT		
miR-1323	Up	TP53INP1	miR-1323 inhibits trophoblast cell viability by inhibiting TP53INP1	Serum HTR-8/SVneo cells BeWo cells	110 GDM	78 NGT	*Homo sapiens*	[73]
miR-152	Up	SOCS3	miR-152 inhibits HIR in GDM mice by downregulating the expression of SOCS3	Blood Islet cells	–		Mouse	[81]
miR-134-5p	Up	FOXP2	miR-134-5p promotes inflammation and apoptosis of trophoblast cells via regulating FOXP2 transcription in GDM	Serum HRT-8/SVneo cells	70 GDM	70 NGT	*Homo sapiens*	[71]
miR-377	Up	FNDC5	miR-377 inhibition enhances the survival of trophoblast cells via upregulation of FNDC5 in GDM	Serum HTR-8/SVneo cells BeWo cells	30 GDM	38 NGT	*Homo sapiens*	[72]
miR-875-5p	Down	TXNRD1	miR-875-5p silencing notably reduces fasting blood glucose and insulin resistance	Serum	–		Rat	[83]

miRNAs, microRNAs; GDM, gestational diabetes mellitus; NGT, normal glucose tolerance; hPMECs, human placental microvascular endothelial cells; HUVECs, human umbilical vein endothelial cells; MUVECs, mouse umbilical vein endothelial cells; PBMCs, peripheral blood mononuclear cells; IR, insulin resistance; IS, insulin sensitivity; EVs, extracellular vehicles; HIR, hepatic insulin resistance.

miRNA can regulate the function of tissues and cells in GDM patients by targeting mRNAs, genes, and activating or inhibiting relevant pathways, thus participating in the pathogenesis of GDM. For example, the expression of 48 miRNAs, including miR-574-5p and miR-3135b, was significantly reduced in plasma samples from GDM patients as compared to healthy pregnant women. The expression levels of these miRNAs and the target genes they work with are associated with glucose and lipid metabolism, as well as insulin signaling pathways [62]. miR-518 is highly expressed in the serum of patients with GDM and GDM complicated with HDCP (GDM&HDCP). This miRNA can inhibit the regulation of inflammatory factors by peroxisome proliferator-activated receptor α (PPARα) [63]. Indeed, the critical functions of trophoblast cells during pregnancy have been elucidated, and extensive research suggests that miRNAs can regulate trophoblast cell function through various mechanisms. A study of miRNA dysregulation in placental exosomes found that miR-135a-5p expression was significantly upregulated in placenta-derived exosomes from pregnant women with GDM. miR-135a-5p activated the PI3K/AKT signaling pathway by targeting Sirtuin 1 (SIRT1) and thus increased the proliferation, invasion and migration of placental trophoblast cells [64]. Upregulation of miR-195-5p expression was found in an *in vitro* model of human placental microvascular endothelial cells (hPMECs) treated with high concentrations of grapes. miRNAs not only regulate proliferation, apoptosis and angiogenesis of hPMECs, but also regulate GDM by targeting VEGFA apoptosis and pathological changes of placental tissues in mouse models *in vivo* [65]. However, miR-362-5p, which is expressed at a low level in placental tissues of GDM patients, affects the PI3K/AKT pathway and apoptosis-associated factors by negatively regulating the target gene GSR, thereby inhibiting the proliferation and promoting apoptosis in HG-treated HTR-8/SVneo cells [66]. Similarly, miR-30d-5p expression is downregulated in the placenta of GDM patients as compared to normal controls, and it binds to RAB8A mRNA to inhibit the expression of this gene, which impairs trophoblast cell function [67]. In placental tissues, miR-17-5p expression is identically downregulated, which improves glucose uptake by HTR-8/SVneo cells by targeting TXNIP and NLRP3 [68]. In addition, Li et al. reported for the first time that miR-22 expression was significantly downregulated in placental tissues of GDM patients, along with miR-372, whose expression levels were negatively correlated with HG exposure. *In vitro* studies demonstrated that these two miRNAs could target SLC2A4, the gene encoding GLUT4, in order to regulate its transcription or to influence insulin signaling pathways by stabilizing the GLUT4 translation or degradation of the transcript. This, in turn, affects the insulin signaling pathway in GDM [69]. Downregulation of miR-143-3p levels was found in plasma samples from 30 pairs of GDM patients and healthy women. Overexpression of miR-143-3p in HG-treated MIN6 cells showed that it inhibited the TAK1/NF-κB signaling pathway to promote cell viability and insulin secretion and prevent pancreatic β-cell dysfunction [70]. Dysregulation of miRNAs in serum may also regulate trophoblast function through genes and related pathways. Serum miR-134-5p was elevated in patients with GDM compared to healthy pregnant women and was found to exacerbate GDM by mediating trophoblast inflammation and apoptosis through regulation of FOXP2 transcription in HTR-8/SVneo cells [71]. Similarly, serum miR-377-3p was elevated in patients with GDM, and miR-377-3p was found to directly target FNDC5 in a cell model, promoting GDM by inhibiting cell growth reconstitution and increasing the rate of apoptosis [72]. In addition, elevated serum levels of miR-1323 inhibits the expression of TP53INP1, which reduces trophoblast cell viability and leads to hyperglycemia [73]. Some studies have also focused on the expression changes and mediating pathways of miRNAs in HUVEC cells. For example, miR-34b-3p was upregulated in HUVECs from GDM patients, and it was found to impair HUVEC viability and migration in GDM by targeting PDK1 in *in vitro* simulated GDM [74]. In the study of exosomal miRNA levels in the placenta-derived mesenchymal stem cells (PlaMSCs) from GDM patients (GDM-MSCs), elevated expression levels of miR-130b-3p were found. Subsequent studies in GDM mice confirmed that miR-130b-3p regulates ICAM-1 expression, inhibiting HUVEC proliferation, migration and angiogenesis [75]. miR-6869-5p was considerably downregulated in placenta-derived macrophages from GDM patients. It prevents macrophage from targeting PTPRO and promotes macrophage polarization to M2-type cells, thus preventing macrophage proliferation and inflammation, and maintaining the balance of the placental microenvironment [76]. In addition, one study identified 22 dysregulated exosomal miRNAs in the plasma of pregnant women with GDM and verified that upregulated miR-423-5p and downregulated miR-122-5p, miR-148a-3p, miR-192-5p and miR-99a-5p could be used as early predictors of GDM. Among them, miR-122-5p may be involved in GDM metabolic regulation with targeting G6PC3 and FDFT1 genes to regulate the insulin and AMPK signaling pathways [77]. Notably, miRNAs can also regulate GDM through some more interesting mechanisms. For example, the expression level of miR-199a-5p is significantly upregulated in the placenta of patients with GDM compared to normal pregnant women. miR-199a-5p can regulate the glucose pathway by repressing Methyl CpG Binding Protein 2 (MeCP2) and downregulating the classical transient receptor potential 3 (Trpc3) expression to regulate the glucose pathway. This suggests that miR-199a-5p may regulate the glucose pathway by modulating methylation levels, leading to GDM [78]. Zeng et al. investigated the association between polymorphisms of miR-196a2 and miR-27a and susceptibility to gestational diabetes mellitus in Chinese population, and found that miR-196a2 rs11614913 and miR-27a rs11614913 and miR-27a variants may negatively regulate lipocalin gene expression and increase susceptibility to GDM [79].

In addition to studies based primarily on samples from GDM patients, analyses have also been conducted on the expression levels and mechanisms of miRNAs in mouse and rat models of GDM. In mouse C2C12 cells, miR-182-3p expression was significantly upregulated. miR-182-3p inhibitor can directly bind to INSR1, a key regulator of the insulin-related pathway, and increase the expression of NSR1 and its downstream signaling pathway in skeletal muscle, thereby promoting GLUT4 translocation as well as glucose uptake and utilization, and thus alleviating the development of GDM [80]. In mouse pancreatic islet tissues, miR-152 inhibits hepatic insulin resistance (HIR) in GDM mice by downregulating the expression of cytokine signaling 3 (SOCS3) [81]. Similarly in pancreatic tissues, miR-210-3p is significantly overexpressed, which can directly target Dtx1 and negatively regulate its expression to accelerate the development of GDM, thereby damaging glandular β-cell function and cell viability [82]. Studies on pregnant rats found that miR-875-5p expression level was downregulated. miR-875-5p can regulate IR and inflammation through TXNRD1. Meanwhile, silencing miR-875-5p significantly reduced fasting blood‑glucose and insulin resistance, lowered the expression levels of lipids and pro-inflammatory markers, and decreased oxidative stress levels [83].

### Circular RNAs

Although research on the regulatory mechanisms of circRNAs is relatively limited at present, existing studies have already demonstrated their significant role in the pathogenesis of GDM (Table 3). Variations in circRNA expression levels can be used to predict GDM. A study on the expression levels of plasma exosomal hsa_cirRNA_0039480 in early, mid and late pregnancy in patients with GDM found that this circRNA showed high expression at all three stages [84]. In plasma samples, Zhu et al. found that actin-related protein 2 homolog (circACTR2) is overexpressed in GDM, and high plasma levels of circACTR2 are closely associated with adverse events such as preterm birth, miscarriage and fetal malformation [85]. Similarly, circVEGFC is upregulated in the plasma of GDM patients. Receiver operating characteristic curve analysis shows that the high expression level of circVEGFC on the day of admission exhibits higher sensitivity and specificity for the early diagnosis of GDM [86, 87]. A retrospective case–control study found that the expression level of hsa_circ_102682 was lower in GDM patients than in the control group, and was significantly correlated with triglycerides, apolipoprotein A1 (APOA1), apolipoprotein B (APOB) and 1 h blood glucose. These results suggest that hsa_circ_102682 may regulate lipid metabolism to participate in the pathogenesis of GDM [88]. In addition, Zou et al. found that the expression of hsa_circ_0003218 was dramatically downregulated in the GDM group. hsa_circ_0003218 was significantly correlated with the GDM risk factor 25(OH)D3, and the two may be jointly involved in the metabolic process of GDM. Therefore, their combination can be used as a predictive marker for the early stage of GDM [89].

**Table 3:** Summary and functions of aberrant circRNAs in GDM

circRNAs	Expression in GDM	Target	Function	Source	Groups		Species	Ref.
hsa_circ_0003218	Down	25(OH)D3	25(OH)D3 along with hsa_circ_0003218 serves as biomarkers for early prediction of GDM	Serum	45 GDM	65 NGT	*Homo sapiens*	[89]
circHIPK3	Up	miR-1278 DNMT1	circHIPK3 facilitates ferroptosis via miR-1278/DNMT1 to regulate GPX4 DNA methylation in HG-cultured cells	Placental Plasma HTR-8/SVneo cells	40 GDM	40 NGT	*Homo sapiens*	[95]
circSESN2	Up	IGF2BP2	circSESN2 exacerbates HG-induced trophoblast cell damage by binding IGF2BP2 and upregulating its protein expression	Peripheral blood	GSE1827376 GDM 6 NGT		*Homo sapiens*	[92]
								
circ_0001578	Down	NF-κB and JNK pathways	The downregulation of circ_0001578 promotes GDM by inducing chronic inflammation in the placenta via the NF-κB and JNK pathways	Plasma Placental	60 GDM	60 NGT	*Homo sapiens*	[90]
circFOXP1	Down	miR-508-3p	circFOXP1 promotes the growth and survival of HG-treated human trophoblast cells through the miR-508-3p/SMAD2 pathway	Serum HTR-8/SVneo cells	20 GDM	13 NGT	*Homo sapiens*	[91]
hsa_circ_0039480	Up	–	Plasma exosomal hsa_cirRNA_0039480 serves as a candidate biomarker for early detection of GDMs	Plasma exosomes	**First trimester**		*Homo sapiens*	[84]
					24 GDM	43 NGT		
					**Second trimester**			
					58 GDM	56 NGT		
					**Third trimester**			
					46 GDM	47 NGT		
circMAP3K4	Up	miR-6795-5p	circMAP3K4 suppresses insulin-PI3K/Akt signaling pathway via miR-6795-5p/PTPN1 axis, contributing to GDM-related IR.	Placenta HTR-8/SVneo cells	**Discovery**		*Homo sapiens*	[94]
					9 GDM	9 NGT		
					**Validation**			
					42 GDM	42 NGT		
circCBLB circITPR3 circNFKBIA circICAM1	–	CBLB ITPR3 NFKBIA ICAM1	circCBLB, circITPR3 and circICAM1 serve as GDM-related miRNA sponges and regulate the expression of CBLB, ITPR3, NFKBIA and ICAM1 in cellular immune pathways	PBMCs	**Discovery**		*Homo sapiens*	[97]
					6 GDM	6 NGT		
					**Validation**			
					28 GDM	28 NGT		
hsa_circ_0046060	Up	hsa-miR-338-3p	Exosomal hsa_circ_0046060 derived from UMSC targets G6PC2, affecting glucose homeostasis and inducing IR through hsa-miR-338-3p	Human liver cell line L-02 UMSCs	–		*Homo sapiens* Mouse	[98]
circDMNT1	Up	p53	circDMNT1 induces p53 expression and activates the JAK/STAT signaling pathway	Placenta HTR-8/SVneo cells	35 GDM 35 NGT 35 PE		*Homo sapiens*	[93]
circACTR2	Up	–	The increased plasma circACTR2 levels in pregnant women predicts GDM	Plasma	70 GDM	130 NGT	*Homo sapiens*	[85]
circPNPT1	Up	miR-889-3p	circPNPT1 promotes HG-induced trophoblast cell biological dysfunction through miR-889-3p/PAK1 axis	Placental	19 GDM	19 NGT	*Homo sapiens*	[96]
hsa_circRNA_102682	Down	–	hsa_circRNA_102682 participates in the regulation of glucose metabolism and lipid metabolism	Serum	300 GDM	300 NGT	*Homo sapiens*	[88]
circVEGFC	Up	–	High expression levels of circVEGFC might serve as an early predictor of GDM and its adverse events	Plasma	110 High circVEGFC 110 Low circVEGFC		*Homo sapiens*	[86]
circ_0074673	Up	miR-1200	Exosomal circ_0074673 affects the biological behavior of HG-HUVECs by regulating the miR-1200/MEOX2 axis	Umbilical cord blood exosomes	30 GDM	30 NGT	*Homo sapiens*	[87]

circRNAs, circular RNAs; GDM, gestational diabetes mellitus; NGT, normal glucose tolerance; HG, high glucose; IR, insulin resistance; PBMCs, peripheral blood mononuclear cells; UMSCs, umbilical cord mesenchymal stromal cells; HUVECs, human umbilical vein endothelial cells; PE, preeclampsia; High circVEGFC, high expression level of circVEGFC; Low circVEGFC, low expression level of circVEGFC.

Moreover, circRNAs can be involved in GDM-related biological processes in multiple ways, including binding to genes or proteins, activating related signaling pathways or acting as miRNA sponges. In studies exploring the effects of HG on trophoblast cells, downregulation of circ_0001578 may promote GDM by inducing chronic inflammation in the placenta via NF-κB and JNK pathways [90]. Li et al. first revealed that the decreased expression of circFOXP1 induces damage to trophoblast cells by regulating the expression of miR-508-3p and downstream SMAD2 molecules in the pathological state of GDM *in vitro* [91]. In addition, the high expression of circRNAs also has an impact on cellular functions. circSESN2 is overexpressed in patients with GDM and exacerbates HG-induced trophoblast cell injury via binding to IGF2BP2 and upregulating its protein expression [92]. Similarly, in the GDM group, circDNMT1 is found to be overexpressed. It mainly inhibits trophoblast cell viability, migration and invasion, and induces cell apoptosis and cell-cycle arrest by binding to p53 and activating the JAK/STAT signaling pathway [93]. In addition, circMAP3K4 can regulate the expression of PTPN1 by binding to miR-6795-5p, thereby modulating the insulin-PI3K/Akt signaling pathway and inhibiting glucose uptake in trophoblast cells. This regulatory mechanism may contribute to IR associated with GDM [94]. Notably, circRNA might affect the level of DNA methylation. In GDM, circHIPK3 binds to miR-1278 and targets DNM1. The high expression of circHIPK3 affects the methylation status of the GPX4 gene through this molecular pathway, leading to ferroptosis in HTR-8/SVneo cells under HG culture conditions [95]. Besides, circPNPT1 can directly sponge miR-889-3p to promote the expression of miR-889-3p targeted PAK1. The high expression of circPNPT1 in GDM patients can promote cellular biological dysfunction through the miR-889-3p/PAK1 axis [96]. circCBLB, circITPR3 and circICAM1 may also serve as GDM-related miRNA sponges and regulate the expression of CBLB, ITPR3, NFKBIA and ICAM1 in cellular immune pathways [97]. Knockdown experiments of circ_0074673 facilitated the proliferation, migration and angiogenesis of high glucose-treated human HUVECs via acting as a sponge for miR-1200. This finding may provide a potential target for the treatment of GDM. Notably, circRNAs in GDM exosomes may also serve as potential biomarkers and therapeutic targets for GDM. hsa_circ_0046060 in exosomes derived from hUMSC regulates glucose homeostasis and induces insulin resistance in normal human liver cell L-02 and GDM mice by targeting G6PC2 via hsa-miR-338-3p [98].

## DISCUSSION

Most of the human genome is transcribed into ncRNAs, which can regulate numerous physiological, developmental and disease processes, holding significant potential as therapeutic targets for diseases. The development of RNA sequencing technologies has led to the discovery of a growing number of ncRNAs, laying a solid foundation for researchers to investigate the regulation mechanisms of ncRNA. To date, a substantial amount of research has been published on how dysregulated ncRNAs participate in the development of various diseases. This article provides a summary of the regulatory mechanisms of lncRNAs, miRNAs and circRNAs in cases on GDM. ncRNAs may target genes, mRNAs or proteins and regulate their transcription and translation, to affect downstream signaling pathways related to the development of GDM, such as glucose and lipid metabolism and insulin signaling. Furthermore, ncRNAs may function by modulating methylation levels and forming regulatory networks. These molecular mechanisms could lead to the occurrence of β-cell dysfunction, insulin resistance, adipose tissue dysfunction, gluconeogenesis and oxidative stress, resulting in the development of GDM. The literature described herein provide strong evidence that ncRNAs play important roles in GDM. However, most of these studies are based on biological experiments, typically time consuming and costly, to analyze the regulatory mechanisms of ncRNAs. Therefore, there is an urgent need to find more cost-effective bioinformatics methods to discover potential biomarkers and mechanisms, providing validation insights for biological experiments. This will also help reduce the use of invasive clinical detection methods and improve the diagnostic accuracy of various diseases. A large number of traditional machine learning (ML)-based models, such as random forest (RF), support vector machine (SVM) and logistic regression (LR), have been used to predict biomarkers for a variety of diseases, including GDM. For example, Yoffe et al. constructed RF, LR and AdaBoost models using the levels of significantly upregulated miR-223 and miR-23a in GDM patients as features. These models were used for classifying GDM and healthy women, resulting in favorable classification outcomes [99].

Due to the exponential growth of biological data, deep learning methods have been widely used in various biological fields, such as protein structure and function prediction and disease marker prediction. Naseer et al. constructed a deep neural network with a generic pseudo amino acid composition, called iGluK-Deep, to identify lysine glutamylation sites. They applied a basic quantitative encoding of Pseudo Amino Acid Composition sequences to generate a baseline dataset consisting of strings of integers. Then the dataset was fed into using well-known neural network architectures (DNNs) such as fully connected neural networks (FCNs), convolutional neural networks (CNNs) and recurrent neural networks with simple units, gated recurrent unit and long short-term memory units, respectively. Also, the FCN model showed the higher performance of proposed approach for lysine glutarylation site prediction [100]. Understanding antigen–antibody binding interactions can help in the design of antibodies, therapeutic drugs and vaccines. A novel deep learning model, called DeepBCE, was developed to predict immunostimulatory factor B-cell epitopes from protein sequences to understand the binding mechanisms between antigens and antibodies. This model was developed using a combination of deep CNNs and A position and AA composition variant feature-based feature vectors, and was able to accurately predict linear B-cell epitopes [101]. In addition, there are many other deep learning methods that can be applied to biological data. Among the existing deep learning methods, attention mechanisms were first introduced in order to elucidate the mapping between a query and a set of key–value pairs to an output, where the query, keys, values and output are all vectors. Specifically, the output was computed by a weighted summation of the values, while the weights were computed through a compatibility function of the query with the corresponding key. In simple terms, the attention mechanism calculates the correlations or weights between different parts of the input data, enabling the model to selectively focus on the relevant information in the input. By introducing the attention mechanism, deep learning models can greatly handle long sequences, resolve dependencies between inputs and be more flexible in weighting different parts. Altogether, it is foreseeable that deep learning models based on attention mechanisms are capable of providing a great potential for biological sequence analysis. To date, attention mechanisms have been applied to predict ncRNA–disease associations and interactions with proteins. For instance, heterogeneous graph attention network framework based on meta-paths for predicting lncRNA–disease associations (HGATLDA) is a novel heterogeneous graph attention network framework based on meta-paths for predicting lncRNA–disease associations. In HGATLDA, first, feature matrices were extracted from the multi-view similarity graphs of lncRNAs and diseases with graph convolutional networks. Second, an attention mechanism was used to assign weights to the feature matrices. Then, all representations were extracted using CNNs and then fed into a stacking ensemble classifier for the prediction purpose. Finally, in the case study, eight out of the top-10 lncRNAs predicted by HGATLDA to be associated with colon cancer have been experimentally validated [102]. This shows that the deep learning model can be utilized to effectively characterize the associations between lncRNAs and diseases, and also shows that the biomarkers obtained from its prediction may be of high value for the diagnosis of diseases. In addition, Han et al. constructed a computational model based on a line graph attention network framework, called ncRPI-LGAT, for predicting ncRNA–protein interactions. They transformed the link prediction task into a node classification task in the line network, and then introduced a line graph attention network framework as means to predict ncRNA–protein interactions. ncRPI-LGAT performed well in terms of the prediction of ncRNA–protein interactions across multiple test sets [103]. Moreover, this method is considered as a useful tool that can provide new insights for subsequent experiments exploring the underlying mechanisms of these interactions.

All in all, future research on the regulatory mechanisms of ncRNAs in disease could involve the following aspects: First, potential ncRNA biomarkers can be identified from large-scale sequencing data with deep learning algorithms, including but not limited to attention mechanisms. In addition, the markers can be predicted for disease relevance and their interactions with genes, mRNAs or proteins. This process plays a crucial role in providing important clues and guidance for the validation of biological experiments. Next, as the expression levels and mechanisms of action of ncRNAs may change before the onset of disease and at different stages of disease development, the clinical preventive and diagnostic potential of ncRNAs should be evaluated. As mentioned above, attentional mechanisms can effectively capture the characteristics of different states, thus providing important information for screening ncRNAs with significant functions. Furthermore, there is a need to explore whether ncRNAs can be used for disease treatment. Using attention mechanism modeling, interaction networks between ncRNAs and drugs can be constructed to identify potential correlations, thus supporting the development of novel targeted therapeutic drugs.

**Key Points**

- ncRNA is expected to serve as a potential biomarker for diseases, providing support for early diagnosis and treatment.
- We provided a comprehensive review on several recent studies in terms of the role of ncRNA regulatory mechanisms in GDM.
- We discussed the potential of deep learning approaches for the prediction of disease markers and pathogenic mechanisms.

## Author Biographies

**Dong Gao** is a master student at the Center for Informational Biology, University of Electronic Science and Technology of China. Her research interests are bioinformatics, machine learning and 3D genome.

**Liping Ren** is an associate professor in Chengdu Neusoft University. Her research interests are bioinformatics.

**Yu-Duo Hao** is a master student at the Center for Informational Biology, University of Electronic Science and Technology of China. His research interests are bioinformatics, epigenetics and aging.

**Nalini Schaduangrat** is a researcher at Center for Research Innovation and Biomedical Informatics, Faculty of Medical Technology, Mahidol University. Her research interests include machine learning, drug discovery and development.

**Xiao-Wei Liu** is a master student at the Center for Informational Biology, University of Electronic Science and Technology of China. Her research interests are bioinformatics.

**Shi-Shi Yuan** is a doctor student at the Center for Informational Biology, University of Electronic Science and Technology of China. His research interests are biological data analysis.

**Yu-He Yang** is a doctor student at the Center for Informational Biology, University of Electronic Science and Technology of China. Her research interests are drug bioinformatics.

**Yan Wang** is an associate professor at the Department of Cardiovascular Medicine, Beijing Hospital. Her research is the areas of cardiovascular disease and diabetes.

**Watshara Shoombuatong** is an associate professor at Center for Research Innovation and Biomedical Informatics, Faculty of Medical Technology, Mahidol University. His research interests include bioinformatics, machine learning, drug discovery and development.

**Hui Ding** is an associate professor at the Center for Informational Biology, University of Electronic Science and Technology of China. Her research is in the areas of bioinformatics and system biology.
